# Engineering Stable Cu-Doped SrTiO_3_ Perovskites for Enhanced Photocatalytic CO_2_ Reduction

**DOI:** 10.1021/acs.inorgchem.5c00796

**Published:** 2025-07-21

**Authors:** Mohammed A. M. Bajiri, Niqab Khan, Julio Cesar Camilo Albornoz Diaz, Waldir Avansi, Douglas Gouvêa, Renato V. Gonçalves

**Affiliations:** † Sao Carlos Institute of Physics, University of São Paulo, IFSC–USP, 13566-590 São Carlos, São Paulo, Brazil; ‡ Laboratory of Nanostructured Multifunctional Materials, Department of Physics, Federal University of São Carlos, 13565-905 São Carlos, São Paulo, Brazil; § Laboratory of Ceramic Process, Department of Metallurgical and Materials Engineering, Escola Politécnica da University of São Paulo, 05508-030 São Paulo, São Paulo, Brazil

## Abstract

Photocatalytic CO_2_ reduction presents a sustainable pathway for renewable energy generation while addressing critical environmental challenges. Strontium titanate (SrTiO_3_) has emerged as a highly efficient wide-bandgap semiconductor photocatalyst for CO_2_ reduction. In this study, we investigate copper-doped strontium titanate (Cu/STO), synthesized via a hydrothermal method, which exhibits a well-defined cubic-like structure. The work focuses on optimizing the CO_2_ reduction process by systematically varying the pH of the reaction medium using HCl and NaOH. Our findings reveal that pH significantly influences product selectivity, with a notable shift from methane (CH_4_) to carbon monoxide (CO) production, highlighting the role of pH in modulating reaction pathways and product distribution. The introduction of pH control substances (such as HCl and NaOH) and their side products can influence selectivity by promoting undesired side reactions. Remarkably, the 2Cu/STO photocatalyst demonstrated exceptional structural stability across all tested pH conditions and maintained a highly stable morphology under neutral (uncontrolled) pH. This study introduces a novel strategy for designing stable and high-performance photocatalysts, emphasizing the critical role of pH control in enhancing both the stability and the efficiency of the photocatalytic CO_2_ reduction process. These insights pave the way for advancing sustainable energy solutions through tailored photocatalytic systems.

## Graphical abstract

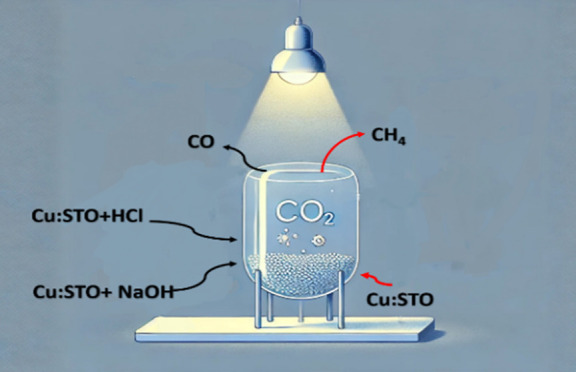

## 1 Introduction

Globally, people consume a significant amount of energy from fossil fuels, which is responsible for approximately 80% of their substantial carbon dioxide (CO_2_) emissions.^1^ The excessive amount of CO_2_ emission to the environment can cause several issues like the greenhouse effect, extreme hurricanes, floods, and droughts.^2,3^ The growing threat of climate change due to increased CO_2_ emissions has encouraged intensive research into efficient methods of CO_2_ capture and conversion.^4,5^ Currently, catalytic technologies are being explored to convert CO_2_ into useful hydrocarbons, including heterogeneous and electrochemical catalysis.^6^ The photocatalysis approach is considered one of the attractive methods for converting CO_2_ into useful products because it can be used at room temperature, and solar light can be utilized as an energy source.^7^ However, the industrial application of photocatalytic CO_2_ conversion remains a difficult challenge, which motivates the search for new materials with enhanced photocatalytic CO_2_ conversion capabilities.^8^ Researchers are exploring various catalyst classes for CO_2_ conversion, including metal oxides,^9^ perovskites, and even exotic materials like MXenes.^10,11^ Perovskite oxides offer remarkable versatility with broad adaptability. This adaptability has made them highly sought-after materials for diverse applications, including catalysis, oxygen transport, and functionalities like ferroelectricity, piezoelectricity, and dielectric properties.^12–15^ Recent research has investigated the potential of certain perovskite materials for photocatalytic applications, including water splitting and CO_2_ reduction.^16,17^ SrTiO_3_ perovskite possesses a theoretical bandgap of 3.24 eV, with both conduction and valence band potentials theoretically suitable for photocatalytic water splitting and CO_2_ reduction.^18,19^ However, the efficiency and product yield of pure SrTiO_3_ in CO_2_ reduction are very low due to its wide bandgap, leading to low visible light utilization and suffering from rapid recombination of photogenerated electron–hole pairs, which reduces its overall photocatalytic activity.^18,20^ To address these issues, researchers are exploring two promising strategies: doping SrTiO_3_ with metals to narrow the bandgap and facilitate visible light absorption and combining it with other semiconductors to improve the separation of photogenerated electron–hole pairs, improve the product selectivity, and extend their lifespan. Wei et al. reported a single crystal of cubes of SrTiO_3_ with high photocatalytic activity, producing CH_4_ up to 4.39 μmol g^–1^ in 8 h but drops to 0.46 μmol g^–1^ beyond 8 h.^21^ Rizzato et al. investigated Cu-doped SrTiO_3_ nanostructured catalysts for photocatalytic CO_2_ conversion into solar fuels, which have demonstrated a high conversion of CO_2_ at 400 °C.^22^ The reaction medium of CO_2_ reduction significantly influences both the product selectivity and stability, with pH being a critical parameter. Different types of compounds have been explored to control the pH, enhance the CO_2_ solubility, and improve the separation efficiency of photogenerated electrons and holes, ultimately impacting the product’s stability and selectivity. It is crucial to understand the type of compounds employed to control the pH and whether these compounds directly interact with the CO_2_ reduction pathway, potentially leading to a change in the product selectivity. Furthermore, investigating the initial products formed on the catalyst surface and the underlying reasons for the change in the selectivity of the products under the different types of pH control compounds is essential for the optimization of CO_2_ reduction processes and more stable products.^23,24^

An indirect Z-scheme Cu-ASTO/Au/CoO*x*-WO_3_ heterostructure exhibited high selectivity and stability for CH_4_ (2.676 μmol cm^–2^ h^–1^) and CH_3_OH (0.517 μmol cm^–2^ h^–1^) production under the presence of NaHCO_3_ (could act as an alkaline buffer, 0.1 M).^25^ Ag/SrNb_2_O_6_ demonstrated a high CO formation rate and selectivity toward CO evolution in aqueous bicarbonate solutions, even without CO_2_ bubbling. Bicarbonate ions (NaHCO_3,_ which could act as a buffer) enhance photoactivity and CO selectivity by promoting the accumulation of carbon-containing species on the photocatalyst surface under a stable pH. The CO_2_ to CO conversion was significantly higher with bicarbonate compared to water without bicarbonate ion, highlighting the crucial role of the containing species and a stable pH in the reaction medium for achieving specific selectivity and stability. In contrast, pH fluctuations during the reaction diminish CO production and negatively impact both selectivity and stability.^26^ Another study showed that the introduction of Cl^–^ ions through salts such as NaCl, KCl, CsCl, MgCl_2_, and CaCl_2_ led to instability in Ni–Al LDH during CO_2_ reduction. While high CO selectivity was observed in the presence of Cl^–^, the subsequent formation of HClO and HCl (interfering species) generated an acidic environment that could negatively influence the selectivity.^27^

Many studies have reported photocatalysts with high selectivity for producing useful hydrocarbons.^28,29^ However, ensuring the long-term stability of these materials remains a challenge. In this study, we synthesized copper-doped perovskite strontium titanate (Cu/SrTiO_3_) via hydrothermal synthesis. The incorporation of Cu ions enhances the structural and morphological properties of SrTiO_3_, thereby improving its CO_2_ photoreduction activity and stability under different pH conditions. Furthermore, we explored how Cl^–^ and OH^–^ ions affect the selectivity and stability of the CO_2_ reduction products. From the investigation of the effect of pH on the photocatalyst stability and selectivity, we aimed to identify an optimal operating environment for CO_2_ reduction. This insight could lead to enhanced catalytic performance and reusability, which are critical factors for advancing CO_2_ reduction technologies.

## 2 Experimental Section

### 2.1 Chemicals

All chemicals were used as received. Strontium nitrate from Sigma-Aldrich (Sr­(NO_3_)_2_, 99%), titanium isopropoxide from Acros (C_12_H_28_O_4_Ti, 98%), copper acetate from Mallinckrodt (Cu­(CO_2_CH_3_)_2_·H_2_O, 98.7%), and the CO_2_ gas from White Martins Company (99.99%). The standard gas to calibrate the gas chromatograph (see the Supporting Information) was from White Martins Company (10% CO_2_, 10% CH_3_OH, 10% H_2_, 40% He, 10% CH_4_, 10% CO, and 10% C_3_H_8_).

### 2.2 Synthesis of SrTiO_3_ and Cu/SrTiO_3_

Initially, a 1.0 M solution of titanium isopropoxide was prepared in 10 mL of isopropanol. Then copper acetate (0, 0.01, 0.02, and 0.05 M) and 1.0 M strontium nitrate were added, and the solution was stirred for 30 min to ensure complete dissolution. Next, approximately 15 mL of a 5.0 M KOH solution was added dropwise while stirring, and the mixture solutions were stirred for an additional hour. The resulting mixture was sealed tightly in an autoclave and subjected to hydrothermal treatment at 200 °C for 4 h using a heating rate of 5 °C min^–1^. The product was washed once with acetic acid and then five times with distilled water to remove any contaminants. Subsequently, the product was dried in an oven at 80 °C overnight. Undoped SrTiO_3_ was synthesized using the same procedure, excluding the addition of copper acetate.^30^ To simplify identification, the resulting products with different Cu-doped dosages to the SrTiO_3_ were labeled as 1Cu/STO, 2Cu/STO, and 5Cu/STO for 0.01, 0.02, and 0.05 M, respectively.

### 2.3 Photocatalytic CO_2_ Reduction

The photocatalytic CO_2_ reduction was conducted in a 100 mL quartz reactor. A 30 mL aqueous suspension containing 10 mg of the catalyst, which was predispersed uniformly using an ultrasonic bath for 5 min, was added to the quartz reactor. The suspension was purged with argon to remove dissolved gases (15 min), followed by evacuation under a vacuum to eliminate residual air. A constant flow of CO_2_ at 3 mL/min was then introduced for 1 h to saturate the system under darkness, followed by a reduction to 1 mL/min for the remaining 4 h of reaction under a solar simulator. The experiments were conducted by using a 300 W xenon (Xe) lamp equipped with a 1.5 Air Mass (AM) filter to simulate solar irradiation. The irradiation power density was calibrated to 500 mW cm^–2^ by adjusting the distance between the lamp and the reactor, as measured using a detector (Gentec-EO XLP 12-3S-H2-D0 thermopile). The reactor system was connected online to an Agilent 7890B gas chromatograph, and the produced gases were analyzed at intervals of 1 h. The stability tests were conducted in three cycles for each of the following pH levels: 3.3, 5.5–8.3, and 11.3. In each cycle, to keep the pH stable, sodium hydroxide (NaOH) and hydrochloric acid (HCl) were added to control the pH of the photocatalytic solution. For the reaction without pH adjustment, the initial pH was 8.3, and after each cycle, the pH naturally decreased to pH 5.5.

## 3 Results and Discussion

### 3.1 Structural and Morphological Properties

The XRD analysis (Figure 1a) confirms the successful formation of both undoped SrTiO_3_ and copper-doped SrTiO_3_ samples. All diffraction peaks were indexed using the standard reference data from ICSD card No. 19796. Interestingly, the 2Cu/STO sample exhibits the lowest microstrain (0.00087065) compared to the other samples (SrTiO_3_ = 0.00097738, 1Cu/STO = 0.00092833, 5Cu/STO = 0.00093044) (Table S1), which means a more uniform and regular morphology. The uniform and regular morphology leads to a decrease in the aggregation and changes in thermodynamic stability associated with higher Cu dopant levels.^31,32^ This suggests a more ordered crystal lattice, potentially leading to enhanced photocatalytic performance due to improved light absorption and efficient separation of photogenerated electron–hole pairs.^33,34^ It is worth mentioning that the lattice parameters, as shown in Table S1, exhibit only slight changes, which are not considered significant. This is primarily due to the similar ionic radii of Cu and Ti^4+^. In SrTiO_3_, Cu is doped into the lattice, and it substitutes for Ti. The ionic radius of Cu^1+^ (0.77 Å) is relatively close to that of Ti^4+^ (0.605 Å). This small difference in size means that the substitution of Cu for Ti does not significantly distort the lattice, and thus, the unit cell dimensions remain nearly constant. As a result, the substitution of Cu ions does not introduce significant changes in the lattice parameters. Furthermore, the Cu doping concentration is relatively low, and the impact on the lattice parameters will be minimal. At low concentrations, the dopant ions could be isolated from each other, and their effects on the lattice is localized. This reduces the likelihood of collective distortions or phase transitions that could alter the lattice parameters.

**Figure 1:**
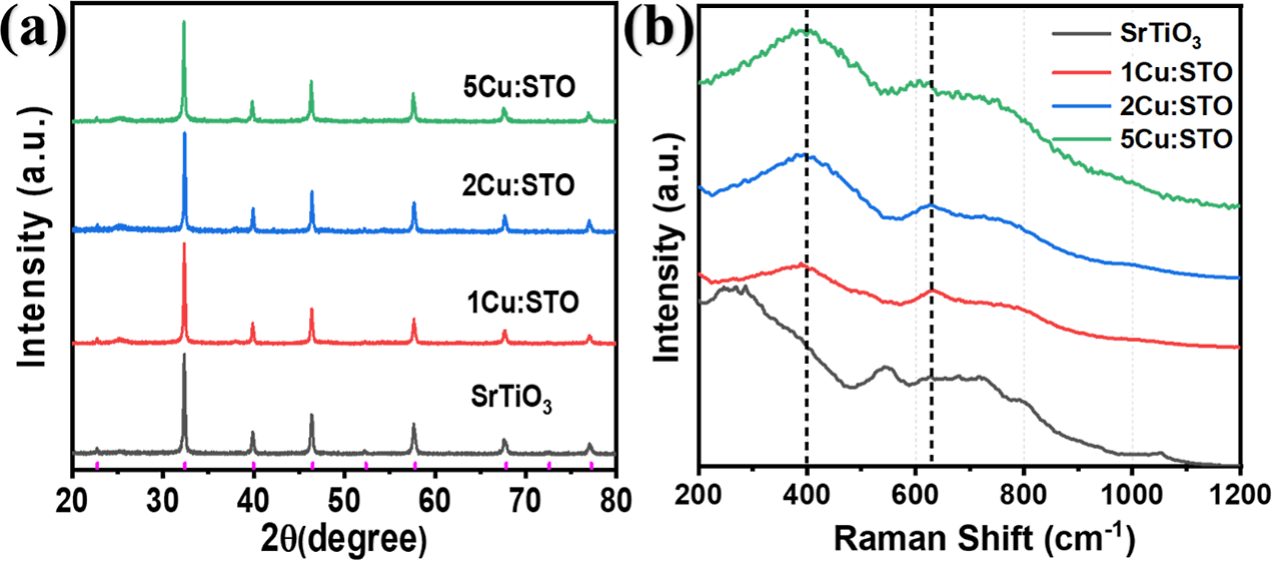
(a) XRD patterns and (b) Raman spectra of SrTiO_3_, 1Cu/STO, 2Cu/STO, and 5Cu/STO.

Raman spectroscopy was employed to investigate the vibrational modes of pure SrTiO_3_, 1Cu/STO, 2Cu/STO, and 5Cu/STO, as shown in Figure 1b. The Raman bands in the 2Cu/STO sample generally appeared slightly sharper compared to the other samples, suggesting a low macrostrain, which is consistent with the XRD results.^35^ By comparing the spectra of pristine SrTiO_3_ and Cu-doped samples, shifts in characteristic band positions (∼400 cm^–1^, and ∼630 cm^–1^) were observed, indicating changes in the bonding environment due to Cu incorporation. The blue shift observed at ∼400 cm^–1^ could potentially be attributed to a distortion of the TiO_*x*_ lattice caused by the presence of Cu,^35,36^ and the red shift observed at ∼630 cm^–1^ might be due to the reduction of Ti by Cu.^35,37^ Raman spectroscopy reveals local distortions around the Cu-substitution sites, which are undetectable by XRD. Despite these localized changes, the lattice parameters remain largely unchanged, according to XRD analysis. Thus, Cu-substitution appears to have minimal impact on the overall lattice structure while inducing local distortions that are evident in the vibrational modes probed by Raman spectroscopy. Therefore, the appearance of this red-shift peak may suggest the formation of new vibrational modes due to Cu-substitution into Ti sites. Moreover, the observed red shift at ∼630 cm^–1^ in 5Cu/STO compared to the other samples is indicative of a Cu_2_O phase as well.^38,39^

To gain insights into the chemical surface composition and electronic structure of the SrTiO_3_, 1Cu/STO, 2Cu/STO, and 5Cu/STO samples, X-ray photoelectron spectroscopy (XPS) analysis was conducted. XPS analysis confirmed the presence of the expected elements, including oxygen, strontium, and titanium, in all Cu-doped SrTiO_3_ samples (1Cu/STO, 2Cu/STO and 5Cu/STO) and SrTiO_3_ (Figure 2). Copper was detected only in the 2Cu/STO and 5Cu/STO samples.

**Figure 2:**
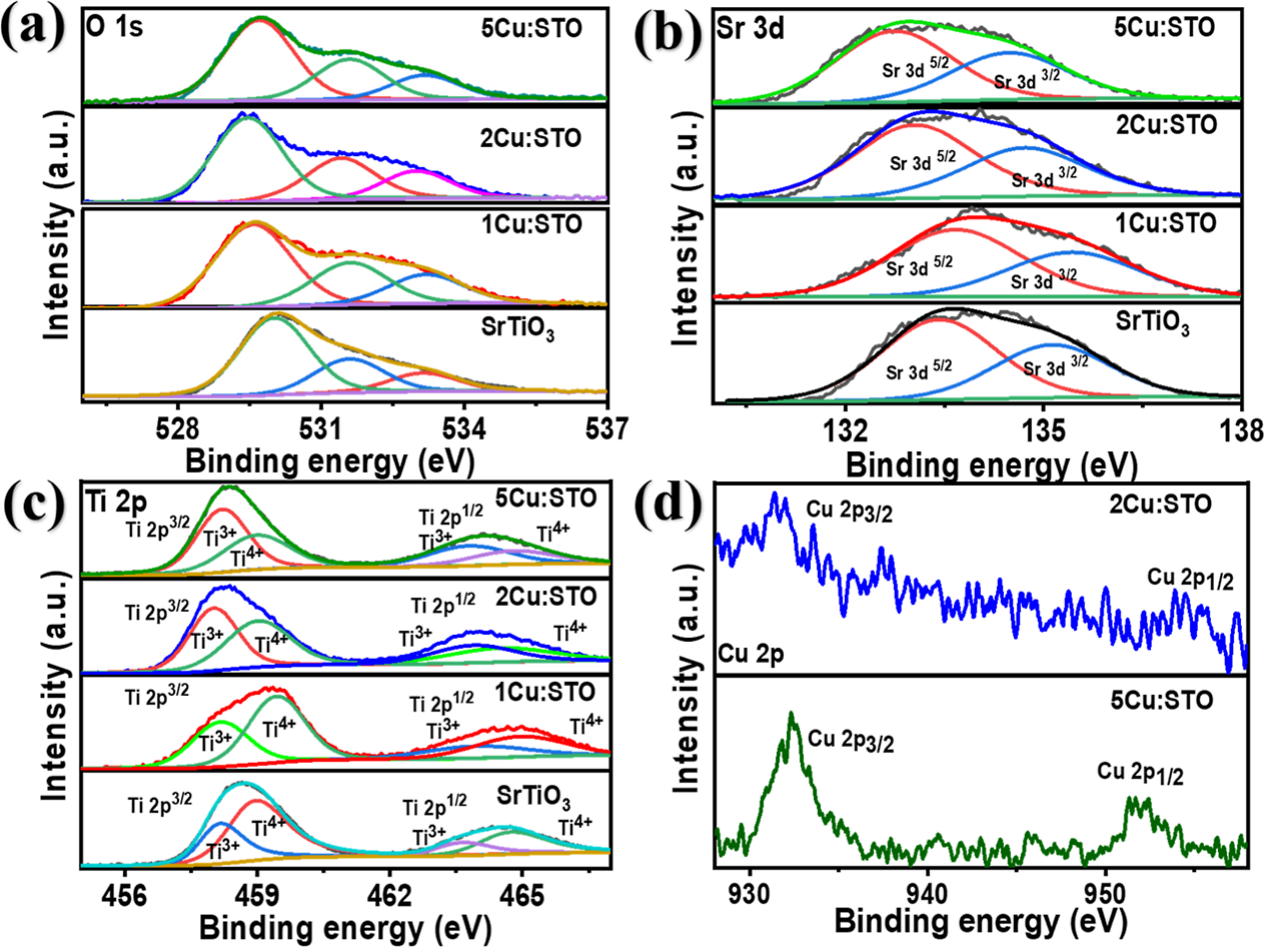
High-resolution XPS spectra of (a) O 1s, (b) Sr 3d, (c) Ti 2p, and (d) Cu 2p of SrTiO_3_, 1Cu/STO, 2Cu/STO, and 5Cu/STO.

The high-resolution O 1s spectra exhibited three peaks for each sample, as shown in Figure 2a. The doped samples showed a low binding energy shift with increasing Cu doping, which could be due to partial Cu doping.^40^ The XPS analysis revealed distinct oxygen-related peaks in the 1Cu/STO and 2Cu/STO samples. Deconvoluted peaks were observed at approximately 530 eV for SrTiO_3_, 529.5 eV for 1Cu/STO, 529.46 eV for 2Cu/STO, and 529.6 eV for 5Cu/STO corresponding to lattice oxygen (O^2–^) within the perovskite structure. These lattice oxygen species are bonded to titanium and strontium ions, playing a critical role in stabilizing the perovskite framework and influencing its structural properties. Additionally, deconvoluted peaks at 531.6 eV for SrTiO_3_, 531.55 eV for 1Cu/STO, 531.4 eV for 2Cu/STO, and 531.61 eV for 5Cu/STO were identified as hydroxyl groups (^−^OH), which serve as active species capable of capturing photoinduced electrons to generate reactive radicals. Furthermore, the peaks at 533.1 eV for SrTiO_3_, 533.2 eV for 1Cu/STO, 533.01 eV for 2Cu/STO, and 533.22 eV for 5Cu/STO were attributed to adsorbed oxygen species, highlighting the complex surface chemistry and potential catalytic activity of these materials. Moreover, 2Cu/STO and 1Cu/STO exhibit a lower binding energy compared to 5Cu/STO. This suggests that a greater amount of Cu was doped on the surface of 5Cu/STO, leading to a higher shift due to interaction with oxygen atoms in the lattice, which could potentially indicate the presence of Cu ions in the structure of SrTiO_3_.^41,42^

Figure 2b shows Sr 3d high-resolution XPS spectra, fitted with two peaks of Sr 3d_5/2_ and Sr 3d_3/2_ with binding energies of approximately 132.0 and 135.2 eV, respectively, corresponding to the Sr^2+^ oxidation state in all samples.^43,44^ In the SrTiO_3_, 1Cu/STO, 2Cu/STO, and 5Cu/STO samples, the Sr 3d_3/2_ peaks were found at 135.1, 135.4, 134.8, and 134.4 eV, respectively.^30,45^ These binding energy shifts indicate the change in the electronic structure of SrTiO_3_ induced by the presence of Cu. Figure 2c presents the Ti 2p core-level XPS spectra for all samples. The observed doublets at 458.4 and 464.0 eV correspond to the Ti 2p_3/2_ and Ti 2p_1/2_ peaks, respectively, which were deconvoluted into two components attributed to Ti^3+^ and Ti^4+^ species. The full width at half-maximum (fwhm) ratio between the Ti 2p_3/2_ and Ti 2p_1/2_ components deviated from the expected 1:1 ratio, indicating asymmetric broadening. This phenomenon is characteristic of the Coster–Kronig effect.^46^ All samples (SrTiO_3_, 1Cu/STO, 2Cu/STO, and 5Cu/STO) exhibited a high-resolution peak at around 458.0 eV, attributed to Ti^3+^, and a broader peak at around 459.0 eV, assigned to Ti^4+^. With increasing Cu concentrations, the surface atomic percentage of the Ti^3+^ increased to 33.3, 40.5, 50.5, and 55.0 atm.%, while that of the Ti^4+^ decreased to 66.7, 59.5, 49.5, and 45.0 atm.%, for SrTiO_3_, 1Cu/STO, 2Cu/STO, and 5Cu/STO, respectively. This suggests that Cu acts as a reducing agent, facilitating the reduction of Ti^4+^ to Ti^3+^.^47^ The reduction effect was observed in 2Cu/STO and was more pronounced in 5Cu/STO, which exhibited the highest Cu doping concentration.

The ionic radius of Cu^1+^ (0.77 Å) is close to that of Ti^4+^ (0.64 Å), facilitating the substitution of Cu into the SrTiO_3_ lattice during synthesis processes.^48^ This substitution creates a deficiency of Ti^4+^ ions, which must be compensated by the reduction of some Ti^4+^ to Ti^3+^ to maintain charge neutrality in the crystal structure.^48^ This likely occurs by balancing the presence of Ti^4+^ and Ti^3+^ ions, making the material more effective for selectivity and photocatalytic applications.^49^ The equilibrium established based on the quantity of Cu doped in SrTiO_3_ could influence the selectivity of CO_2_ reduction, as the interaction through adsorption–desorption processes will occur on the catalyst surface.^50^ Figure 2d displays the detectable Cu peaks in the 2Cu/STO and 5Cu/STO sample peaks at 932.4 and 952.2 eV, corresponding to Cu 2p_3/2_ and Cu 2p_1/2_, respectively. These peaks could be attributed to Cu^+^ or Cu^0^, as their peak positions overlap and cannot be distinctly separated.^51^ Due to the low Cu doping levels in 1Cu/STO, Cu species were not detected by XPS analysis. The absence of copper in the 1Cu/STO sample can be attributed to its relatively low surface concentration or possible segregation at grain boundaries, both of which may fall below the detection limit of the XPS technique.

Therefore, the Cu doping levels in SrTiO_3_ were determined by using an inductively coupled plasma optical emission spectrometer (ICP-OES) (Table S3). This technique confirmed the presence of Cu doping in 1Cu/STO, 2Cu/STO, and 5Cu/STO samples. The measured molar percentages of Cu in the samples were 0.093%, 0.123%, and 0.442%, respectively. The corresponding loss of copper from the initial molar amount introduced was 76.65%, 82.17%, and 74.45%, respectively. This could be due to several reasons, such as the washing method. The first wash used acetic acid, which, combined with the fact that Cu is easily oxidized and the possibility of incomplete doping, could lead to a significant loss of copper. For the 2Cu/STO sample, the molar ratio of Sr/Ti was approximately 1.07 g/100 g, which is closest to the ideal 1:1 ratio. In comparison, 1Cu/STO and 5Cu/STO exhibited ratios of 1.15 and 1.12 g/100 g, respectively.

Field emission scanning electron microscopy (FESEM) was employed to examine the morphological characteristics of the undoped SrTiO_3_, 1Cu/STO, 2Cu/STO, and 5Cu/STO samples, as shown in Figure 3. The FESEM image of the undoped SrTiO_3_ sample reveals a poorly defined morphology composed of aggregated particles. This unique arrangement provides insights into the particle organization and surface features of the undoped material, which serve as a baseline for comparison with the Cu-doped samples. The rough texture observed on the particle surfaces suggests continued particle growth at various stages. For 2Cu/STO, a more uniform morphology was observed by SEM (Figure 3c), which can be attributed to the lowest calculated microstrain (Table S1).

**Figure 3:**
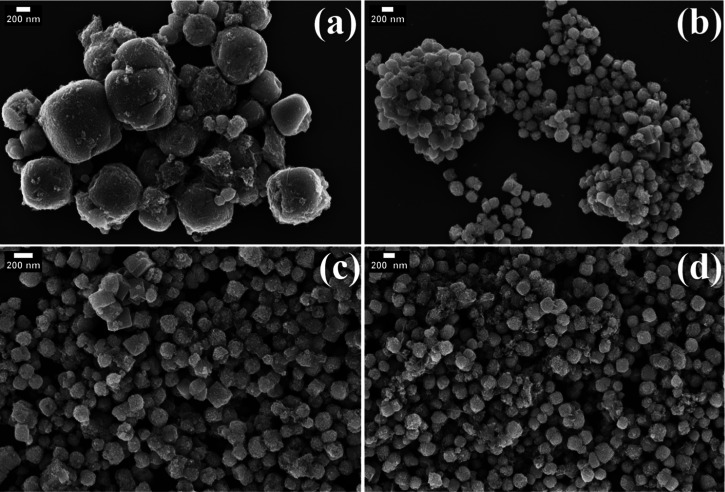
FE-SEM images of (a) SrTiO_3_, (b) 1Cu/STO, (c) 2Cu/STO, and (d) 5Cu/STO.

Representative STEM–EDS elemental mapping (Ti in green, Sr in yellow, and Cu in blue) of 5Cu/STO confirms the presence and homogeneous distribution of Cu ions in the sample (Figure S1). TEM images (Figures 4 and S2) reveal the presence of small particles with a size around 10 nm on 2Cu/STO and 5Cu/STO nanoparticle surface, which explains the rough texture observed on the particles through SEM images (Figure 3). Several studies reported a similar morphological characteristic for the SrTiO_3_ nanostructures (spheres or cubes) even when obtained by different synthesis methods.^52^ The HRTEM image of 2Cu/STO (Figure 4b) shows that the nanocrystal has an interplanar distance equal to 0.36 nm, related to the (100) crystallographic plane, which is in good agreement with the XRD results. For 5Cu/STO, similar HRTEM results could be observed (Figure S2c), where the nanocrystal has an interplanar distance equal to 0.28 nm, related to the (110) crystallographic plane of SrTiO_3_. The HRTEM images indicated in Figure 4c (region B) and their respective fast Fourier transform (FFT) for 2Cu/STO and 5Cu/STO samples (Figures 4c and S2c, respectively) show that the nanocrystals have a single-crystal nature, which occurs due to alignment of the small crystals attributed to the self-assembly crystal growth mechanism, also denoted as oriented attachment crystal growth.^52^ Additionally, HRTEM results do not indicate the presence of any spurious crystalline phase or Cu aggregate in the nanoparticles.

**Figure 4:**
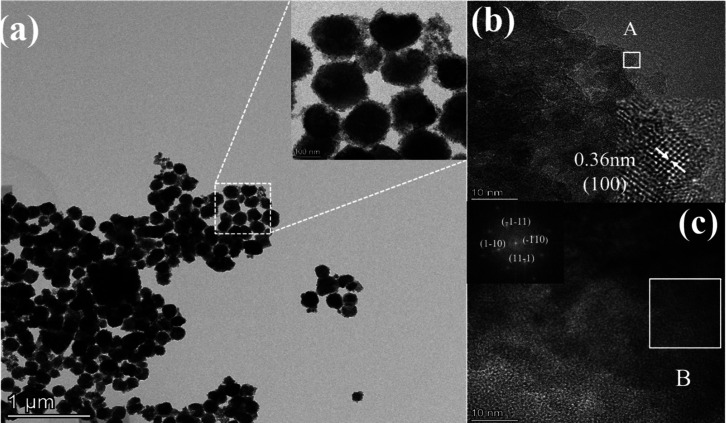
(a) TEM images of 2Cu/STO samples; (b) HRTEM image of nanoparticle surface with an expanded view of region A; (c) HRTEM image of an individual nanoparticle and the respective fast Fourier transform (FFT) of region B (zone axis [112]).

UV–vis analysis was employed within the wavelength range 220–800 nm to assess the bandgap of all the samples. Figure 5a demonstrates that 1Cu/STO, 2Cu/STO, and 5Cu/STO samples exhibit a red-shift light absorption compared to the pure SrTiO_3_.

**Figure 5:**
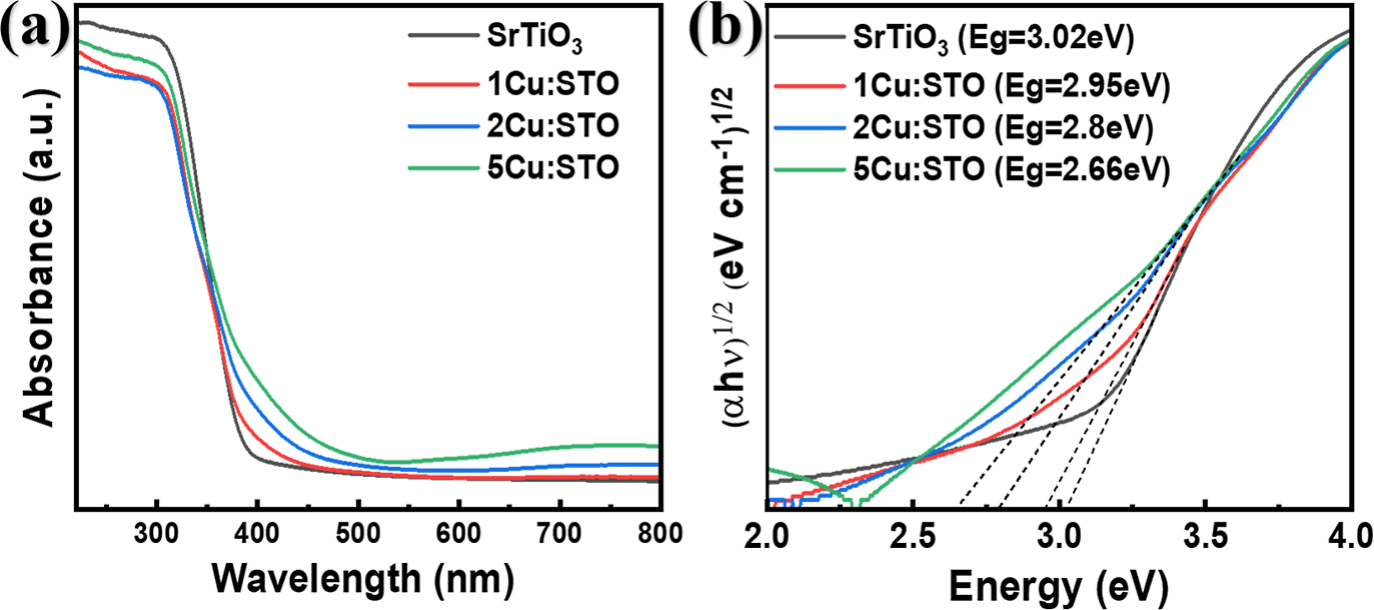
(a) UV–vis absorbance and (b) Tauc plot of SrTiO_3_, 1Cu/STO, 2Cu/STO, and 5Cu/STO, where the bandgap red-shifted from 3.02 to 2.66 eV with the addition of Cu from 0.01 to 0.05 M in SrTiO_3_.

The Tauc plot was used to determine the respective bandgaps of the samples. Figure 5b shows the indirect bandgaps of the samples: 3.02 eV for SrTiO_3_, 2.95 eV for 1Cu/STO, 2.80 eV for 2Cu/STO, and 2.66 eV for 5Cu/STO. As shown in Figure 5b, SrTiO_3_ exhibits a high bandgap, hindering visible light absorption. Conversely, increasing Cu doping leads to a decrease in the bandgap, reaching 2.66 eV for 5Cu/STO.

### 3.2 Photocatalytic CO_2_ Reduction

The photocatalytic efficiency of CO_2_ reduction with SrTiO_3_ and Cu-doped SrTiO_3_ samples was assessed under solar light irradiation and with a CO_2_ flow. Figure 6 exhibits the accumulated CO_2_ reduction products, chiefly CO, CH_4_, and C_2_H_6_, in water. The SrTiO_3_, 1Cu/STO, and 5Cu/STO demonstrated minimal CO_2_ reduction activity with selectivity for CO production. Nevertheless, 2Cu/STO resulted in a 15-fold, augmentation in the CH_4_ production rate and enhanced CH_4_ selectivity compared to the other catalysts (Figure 6 and Table S4). The poor performance of pure SrTiO_3_ is primarily attributed to the large particle size and its wide bandgap (3.02 eV). Conversely, 1Cu/STO and 5Cu/STO show selectivity toward CO production. This could be attributed to the increase of Ti^3+^ and decrease of Ti^4+^ as shown in the XPS analysis and possible partial oxidation of Cu with increasing doping.^53,54^ The optimal Ti^4+^ to Ti^3+^ ratio of almost 1:1 (see XPS) is achieved in 2Cu/STO, which correlates with the highest CH_4_ selectivity. Additionally, control experiments were performed in light and darkness, both with and without the catalyst, and no measurable signs of water-splitting or hydrocarbon products were detected, affirming that the observed CO_2_ reduction products resulted exclusively from the photocatalytic activity of the catalyst.

**Figure 6:**
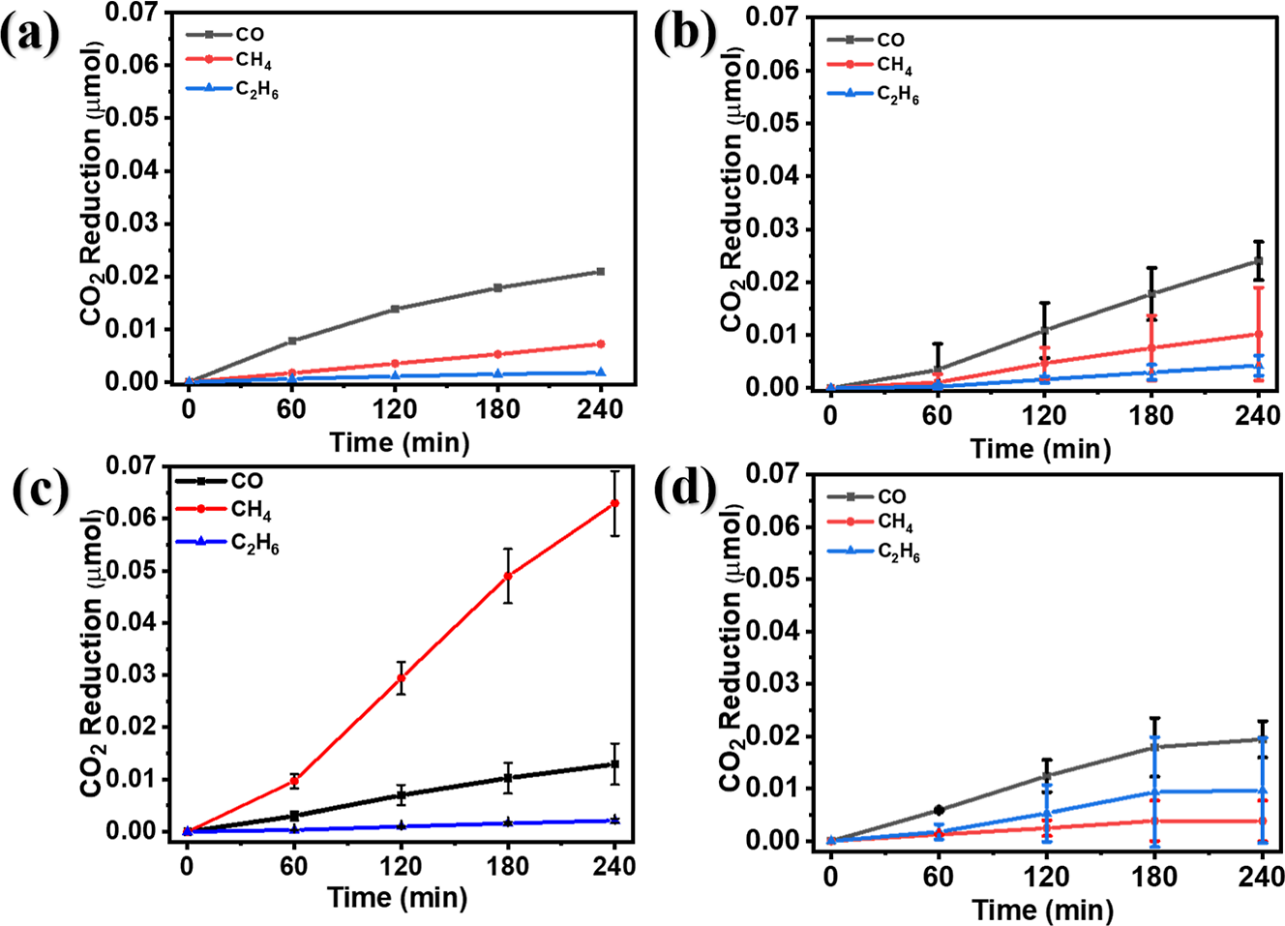
Photocatalytic CO_2_ reduction of (a) SrTiO_3_, (b) 1Cu/STO, (c) 2Cu/STO, and (d) 5Cu/STO.

To assess the stability of the system under specific pH conditions (pH = 3.3, 8.3–5.5, 11.3), the photocatalytic CO_2_ reduction performance of 2Cu/STO was investigated, as depicted in Figure 7. After each cycle, the gas was evacuated from the reactor, and then CO_2_ was injected. This approach was adopted instead of reusability tests to minimize potential sample loss. At pH 3.3 (controlled by HCl), the CO_2_ reduction performance decreased during the cycles, and a change in selectivity from CH_4_ to CO was observed (Figures 7a and 6c). This decreased performance was expected due to the potential side production of CCl_4_ during the photoreduction process (eq 1).

1 $${CO}_{2}+4HCl\to {CCl}_{4}{+2H}_{2}O$$

**Figure 7:**
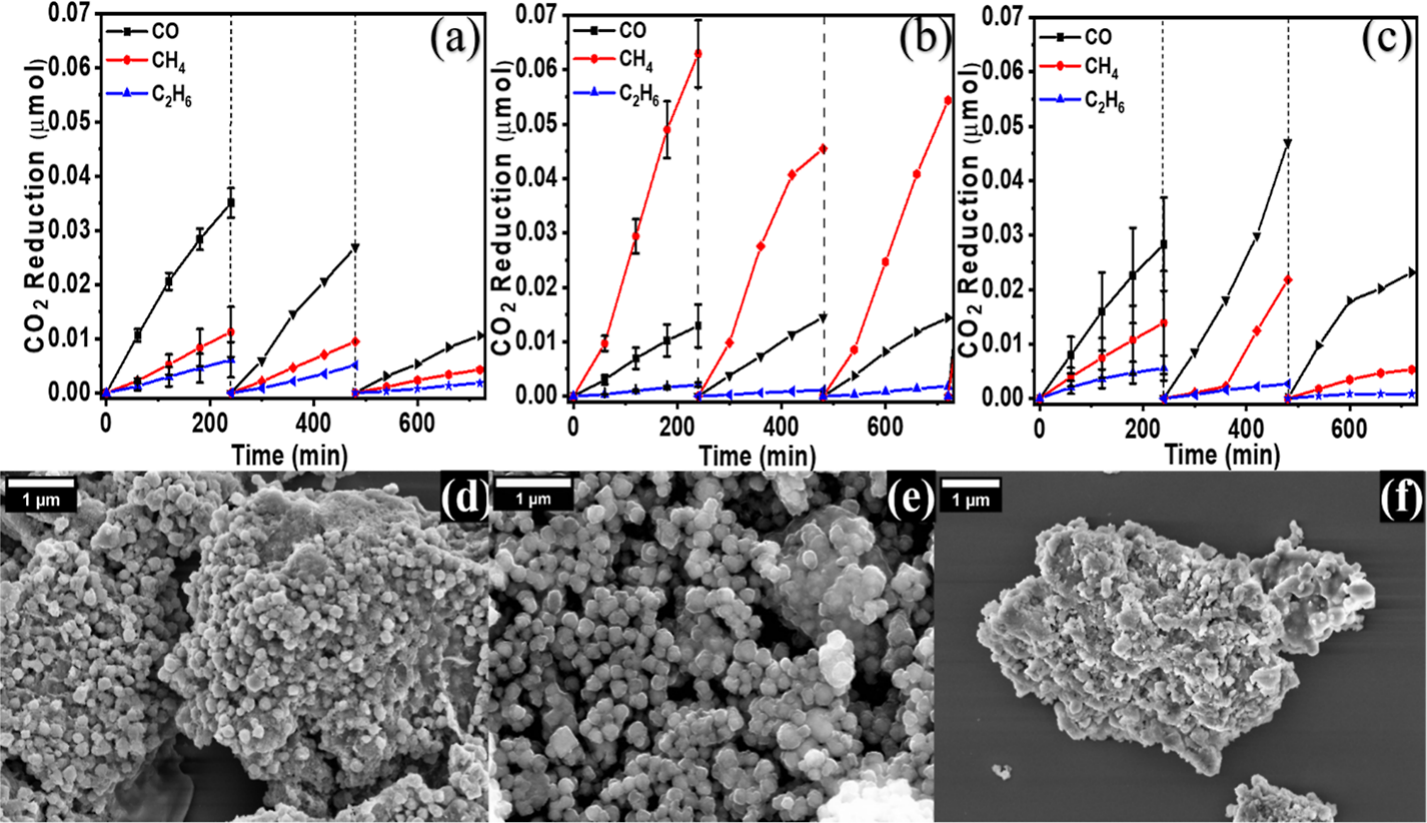
Photocatalytic CO_2_ reduction recyclability of 2Cu/STO with different pH: (a) pH3.3, (b) 8.3–5.5, and (c) pH11.3. The FESEM after the recyclability of 2Cu/STO: (d) pH3, (e) pH8.3–5.5, and (f) pH11.3.

Moreover, nonpolar solvents like CCl_4_, due to their poor solvation properties, can strongly adsorb onto the photocatalyst surface due to their poor solvation properties, which can facilitate CO_2_ reduction to CO and H_2_O. In contrast, it can alter the solubility and availability of CO_2_, potentially reducing the overall efficiency of the photoreduction process.^55,56^ Also, the presence of specific anions like Cl^–^ appears to affect the catalytic selectivity.^27^ Consequently, the selectivity and production rates are also likely to vary depending on the type of acid employed. Indeed, a control experiment involving a photoreaction of CO_2_ with HCl and water, without a catalyst, yielded CO, demonstrating the influence of Cl^–^ on the selectivity of CO_2_ reduction (Figure S3).

This production of CO without a catalyst in the presence of HCl could follow this mechanism:^57^

1-Photolysis of HCl^58^

2 $${HCl+h\nu \to H}^{\cdot }{+Cl}^{\cdot }$$

2-Formation of hydrogen atoms^59^

3 $$H^{\cdot }{+H}_{2}O\to H_{2}{+OH}^{\cdot }$$

3-Reduction of CO_2_ ^55^

4 $${CO}_{2}{+2H\to CO+H}_{2}O$$

At pH 8.3–5.5 (Figure 7b), without any added pH control agents, the highest activity and selectivity for CH_4_ was observed, approximately 6-fold and 3-fold higher than at pH 3.3 (Figure 7a) and pH 11.3 (Figure 7c), respectively. This system also exhibited higher stability compared to those under other pH conditions. However, when the initial pH was 8.3 and pH control with NaOH was implemented after the first and second cycles, a decrease in activity and a shift in selectivity toward CO were observed after the second cycle (Figure S4a), accompanied by significant morphological deformation and agglomeration as shown in Figure S4b. This decline is attributed to the generation of OH^–^ during the pH control process after each cycle, which favored CO production over CH_4_.^60^

The photoreaction rate at pH 11.3 was low due to NaOH (high concentration of OH^–^). Furthermore, the photoreduction of CO_2_ may produce H_2_CO_3_ or CH_3_COOH, as shown in eqs 5 and 6.^55^

5 $${CO}_{2}{+H}_{2}{O\to H}_{2}{CO}_{3}{+OH}^{-}$$

6 $${CH}_{4}{+CO}_{2}\to {CH}_{3}COOH$$

The effects of these acids caused the pH to drop to 5.5 during each cycle, necessitating pH control. These pH variations throughout the cycles, including those controlled at 8.3 via NaOH addition, have the potential to impact both the reaction kinetics and product selectivity due to the introduction of NaOH. Moreover, a control experiment conducted with CO_2_, NaOH, and water alone (without a catalyst) observed a high CO selectivity, highlighting the significant impact of OH^–^ ions within the electrolyte. These ions can form carbonate and bicarbonate, and crucially, they also affect the pH at the surface of the catalyst (in the catalyst present) interface, which in turn influences the selectivity of CO_2_ reduction (Figure S3).^61^ The pH fluctuations during each cycle, including pH 8.3 (controlled with NaOH), can influence the reaction rate and selectivity due to the introduction of NaOH during each cycle.

Conversely, basic conditions can enhance the solubility and adsorption strength of CO_2_ on the catalyst surface, facilitating its conversion to CO, but may also lead to defects in the morphology.^60^ Thus, the pH itself plays a crucial role in determining the catalytic performance as it impacts the reaction environment and the formation of certain reaction intermediates or byproducts.^62^

Figure 7d–f displays the morphology of the samples after three cycles under various pH conditions. The 2Cu/STO sample at pH 8.3–5.5 exhibited the highest stability, showing no significant impact on the morphology compared with other pH conditions, which displayed considerable agglomeration and deformation, particularly at pH 11.3. This phenomenon may be attributed to the pH fluctuations during each cycle, requiring pH adjustments after each cycle. The 2Cu/STO sample at pH 3.3 exhibited agglomeration and minor changes in morphology after the three cycles. While all samples underwent morphological changes, no obvious structural change was observed (Figure S5).

These findings suggest that pH fluctuations can significantly affect the morphology of the catalyst. Furthermore, the recycling method, which involves reusing the same water and accumulated reaction products in each cycle, can contribute to pH fluctuations and affect the overall performance.

### 3.3 Band Position

Ultraviolet photoelectron spectroscopy (UPS) was employed to analyze the band structure of 2Cu/STO photocatalyst, as shown in Figure 8a.^63,64^ The secondary electron cutoff energy (*E*_cutoff_), determined from the UPS spectrum, was used to calculate the work function (Φ), valence band maximum (VBM), and conduction band minimum (CBM) based on eqs 7–11 ^65,66^

7 $$\Phi =h\nu -\left(E_{cut‐off}-V_{bias}\right)$$

8 $$\Phi ={-E}_{F}$$

9 $$VBM=\Phi +E_{VF}$$

10 $$E_{NHE}=-\left(E_{VAC}+4.44\;eV\right)$$

11 $$E_{g}=CBM-VBM$$

**Figure 8:**
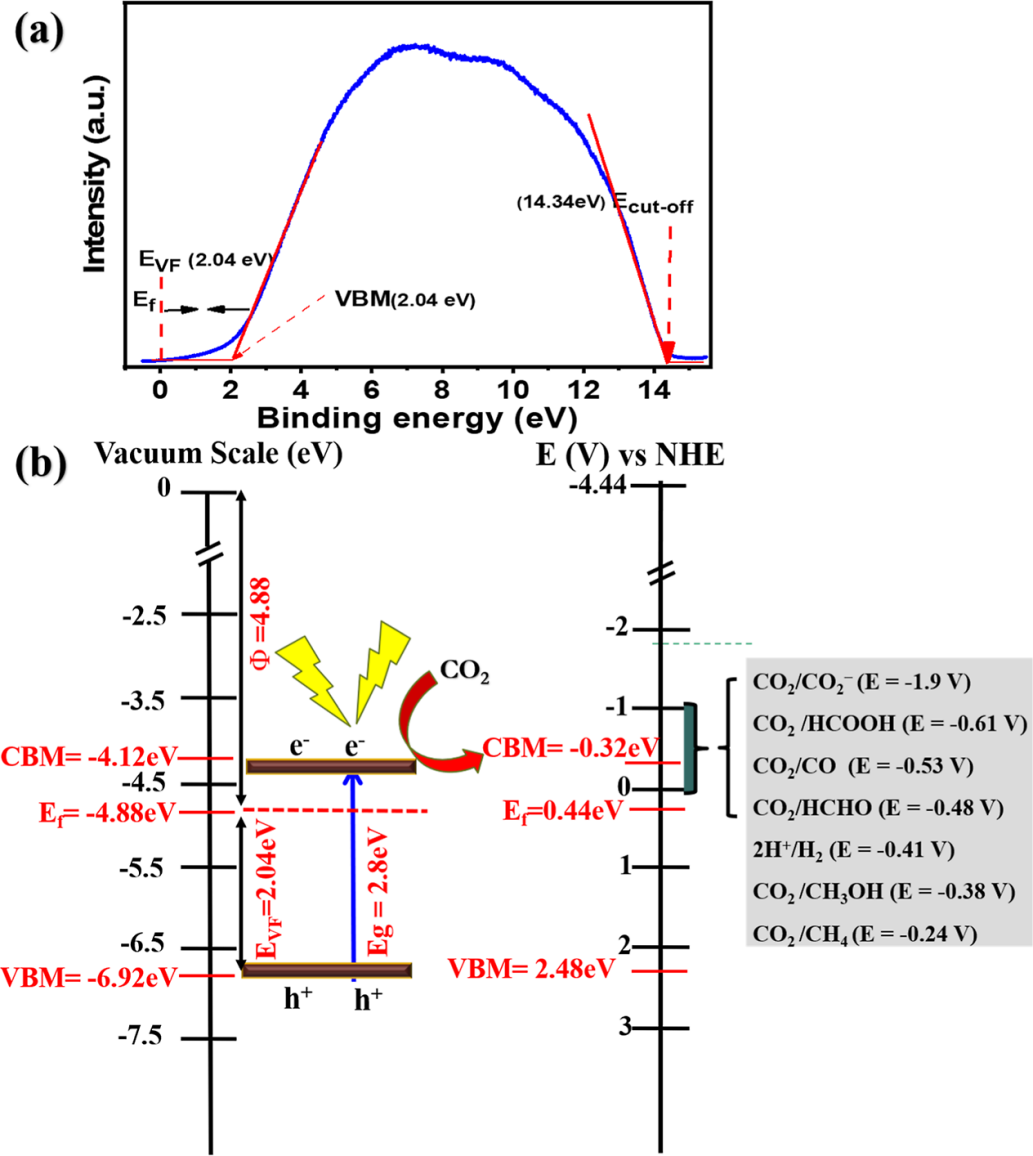
(a) UPS spectrum showing secondary electron cutoff and Fermi edge, with values corrected for −2 V sample bias, and (b) schematic of the charge transfer pathway in 2Cu/STO under illumination.

In eq 7, *h*ν is the photon energy of the He (I) source (21.22 eV), the *E*_cutoff_ for 2Cu/STO was measured as 14.34 eV, and *V*_bias_ is the applied bias voltage (−2 V) during the UPS measurement. Applying eq 7, the work function (Φ) was calculated to be 4.88 eV for the sample of 2Cu/STO. The Fermi level (*E*_F_) was determined from the vacuum level, and the energy in relation to the vacuum level and the normal hydrogen electrode (NHE) level is equal to −4.44 eV (*T* = 298.15 K, pH = 0).^67^

From the UPS data (Figure 8a), according to eq 8, the Fermi level (*E*_F_) corresponds to −4.88 eV. The valence band maximum (VBM) relative to the vacuum level was determined from eq 9 to be −6.92 eV. The *E*_VF_ corresponds to the measured valence band maximum (VBM) relative to UPS spectra, given by its position relative to the Fermi level (*E*_F_), which is conventionally set at zero. Consequently, this value also reflects the energy difference between EF and the VBM, as shown in the graph in Figure 8b.^68^

The energy levels were further converted from the vacuum scale to the NHE scale using eq 10, resulting in a VBM of 2.48 eV (vs NHE). The bandgap (*E*_g_) was determined to be 2.8 eV, as shown in Figure 5b. Consequently, the CBM was calculated to be −0.32 eV (vs NHE) using eq 11. This band structure indicates that 2Cu/STO has sufficient thermodynamic potential to drive photoreduction of CO_2_ to CH_4_, which is consistent with the experimental findings. The formation of CH_4_ likely depends on the surface chemistry, adsorption–desorption behavior, and the presence of Cl^–^ and OH^–^ ions that influence the reaction pathways. In comparison, other samples, such as 1Cu/STO and 5Cu/STO showed variations in their bandgap and Ti^4+^/Ti^3+^ ratios, which subsequently affect their photocatalytic selectivity and performance.

## 4 Conclusion

In conclusion, the 2Cu/STO photocatalyst was synthesized by the hydrothermal method, and its CO_2_ reduction performance was investigated under various pH conditions (3.3, 8.3–5.5, and 11.3). Recycling experiments were conducted to assess the system’s stability and the influence of pH fluctuations on the catalyst’s structure and morphology. At pH 3.3, the CO_2_ reduction performance decreased due to the potential formation of CCl_4_, which can adversely affect the photocatalytic process. Using nonpolar solvents like CCl_4_ can further complicate the reaction by altering the solubility and availability of CO_2_. Further investigation is needed to identify alternative acids that avoid CCl_4_ formation to mitigate these issues. At pH 8.3–5.5, the highest activity and selectivity toward CH_4_ were observed. The higher stability of the catalyst at this pH compared to other conditions suggests that pH changes can affect the photocatalytic activity rate due to increased CO_2_ solubility. Additionally, the introduction of pH control substances (such as HCl and NaOH) can influence the selectivity by promoting side reactions. However, pH fluctuations during the reaction, particularly at pH 11.3, can negatively impact the morphology and the performance. Implementing a buffer solution to maintain a stable pH could be a potential strategy to enhance the stability and efficiency of the system. The pH of the reaction environment plays a crucial role in the photocatalytic CO_2_ reduction performance and selectivity, as does the choice of substance used to control the pH. Optimizing pH conditions and considering potential side reactions can significantly improve the overall efficiency and stability of the system. Further research is needed to identify the optimal pH and buffer solution for enhanced performance and high activity.

## Supplementary Material



## Notes

Characterization data; calculations of crystal size, microstrain, and lattice parameters; STEM, TEM, and HRTEM images of 5Cu/STO; results from inductively coupled plasma optical emission spectrometry; control experiments, table comparing CO_2_ reduction; photocatalytic CO_2_ reduction recyclability of 2Cu/STO with pH 8.3 controlled; XRD after recyclability; and electrochemical impedance measurements (PDF)
