# Supplementary material for: Engineering Stable Cu-Doped SrTiO3 Perovskites for Enhanced Photocatalytic CO2 Reduction
Source: Inorg Chem. 2025 Jul 21;64(30):15370–80. doi: 10.1021/acs.inorgchem.5c00796 (PMC12326357; doi:10.1021/acs.inorgchem.5c00796)
Supplement: Supplementary file 1 [file ic5c00796_si_001.pdf]

Supporting Information

Engineering Stable Cu-Doped $SrTiO_3$ Perovskites for Enhanced Photocatalytic CO_2 Reduction

Mohammed A. M. Bajiri[1], Niqab Khan[1], Julio Cesar Camilo Albornoz Diaz[1], Waldir Avansi Jr.[2], Douglas Gouvêa[3], Renato V. Gonçalves[1*]

[1]Sao Carlos Institute of Physics, University of Sao Paulo, IFSC – USP,

13566-590, Sao Carlos, SP, Brazil.

[2]Laboratory of Nanostructured Multifunctional Materials, Department of Physics, Federal

University of São Carlos, 13565-905, São Carlos, SP, Brazil.

[3]Laboratory of Ceramic Process, Department of Metallurgical and Materials Engineering, Escola

Politécnica da University of Sao Paulo, 05508-030, São Paulo SP, Brazil.

***Email: rgoncalves@ifsc.usp.br**

1. Characterization

X-ray diffraction (XRD) was used to investigate the structural properties of Cu-Doped $SrTiO_3$ and $SrTiO_3$ (Rigaku Ultima IV diffractometer) with a Cu Kα radiation with 2θ angle from 10° to 80° and a step scan of 0.02°. Scanning electron microscopy (FESEM) was used to investigate the morphology of the samples (Zeiss Sigma Gemini model field emission microscope operating at an accelerating voltage of 20 kV). The as-obtained samples also were analyzed through Transmission Electron Microscopy (TEM) and Scanning Transmission Electron Microscopy (STEM) performed on Thermo Scientific Talos F200 (operating at 200 kV) equipped with a Super-X EDS (energy dispersive X-ray spectroscopy) system for chemical analysis. UV-vis absorption measurements of the pure $SrTiO_3$ and $SrTiO_3$ doped Cu were performed using a Shimadzu UV-2600 spectrometer fitted with an integrating sphere. Surface composition and the electronic band structure of materials (work function and the valence band) were analyzed by X-ray photoelectron spectroscopy (XPS) and ultraviolet photoelectron spectroscopy (UPS) using a Scienta-Omicron ESCA+ spectrometer with a monochromatic Al Kα ($h\nu$ = 1486.6 eV) radiation source. The XPS spectra were recorded at a pass energy of 30 eV with a 0.05 eV per step. Charging effects were eliminated using a low-energy electron flood gun. Adventitious carbon (C1s) at 284.8 eV was used

to calibrate the XPS spectra. Data analysis was performed using CasaXPS software using a Shirley-type background subtraction before the curve fitting. The light source was a 500 W Cermax Xe lamp, and the irradiation power density was calibrated using a Gentec-EO XLP 12-3S-H2-D0 photometer by adjusting the lamp-ballast distance. The reactor system was connected to an Agilent 7890B gas chromatograph, which automatically injected gas at intervals and measured the micromolar volumes of H_2. The photocatalytic reactor was developed at the Artificial Photosynthesis Laboratory of the NaCA group at the São Carlos Institute of Physics at USP. The levels presented were determined in a sample prepared by fusion with sodium tetraborate and dosed in an Inductively Coupled Plasma Optical Emission Spectrometer (ICP OES), Horiba brand, model Ultima Expert.

Table S1: Crystal size, micro-strain, and lattice parameters.

Samples	2θ	θradians	FWHM (β)	FWHM (radians)	Cosθ	Sinθ
$SrTiO_3$	32.33	0.282132	0.23322	0.004070457	0.960464	0.278404
1Cu:STO	32.39	0.282656	0.22155	0.003866777	0.960318	0.278907
2Cu:STO	32.42	0.282918	0.2078	0.003626794	0.960245	0.279159
5Cu:STO	32.36707	0.282456	0.22204	0.003875329	0.960374	0.278715

Samples	d=λ/2sinθ	Crystallite Size	Micro-strain (ε)	a=b

$SrTiO_3$	2.76576	35.46337	0.00097738	3.2543
1Cu:STO	2.760774	37.33705	0.00092833	3.2638
2Cu:STO	2.758288	39.81065	0.00087065	3.2582
5Cu:STO	2.762677	37.25249	0.00093044	3.2534

* a and b are lattice parameters.

A 2θ value, where θ represents Bragg's diffraction angle, corresponding to the (**hkl**=110) plane was obtained from the XRD data. The full width at half maximum (FWHM) of the XRD peaks was converted to radians. The micro-strain (ε) x 10^{-3} was then calculated by the FWHM in radians and Cosθ with the following formula(S1):

$$\textbf{Micro-strain } (\varepsilon) 10^{-3} = \left(\frac{\text{FWHM radian} * \text{Cos}\theta}{4}\right) 10^{-3} \qquad \text{(S1)}$$

The crystal size had been calculated using the following formula(S2):

$$\textbf{Crystallite size} = \frac{0.9\,\lambda}{\text{FWHM Cos}\theta} \qquad \text{(S2)}$$

Where the wavelength (λ) is 1.54 Å.

The lattice constants **a** and **b** were calculated using the following formula(S3):

$$\boldsymbol{d} = \frac{\mathbf{1}}{\sqrt{4/3(h^2+hk+k^2)/a^2+l^2/c^2}} \qquad \text{(S3)}$$

Where d is the d-spacing in angstroms (Å)[1].

For the 2Cu:STO (100) peak at $2\theta=22.78^{o}$, the d-spacing is 0.389 nm.

2. Band structure studies

The Tauc plot, determined using equations 1 and 2, was used to identify the respective bandgaps of the catalyst.

$$(\alpha h\nu)^{m} = K(h\nu - E_g)^2 \quad \text{(S4)}$$

$$E_g = 1240/\lambda \quad \text{(S5)}$$

The α, $h\nu$, K, E_g, and λ indicate the absorption coefficient, photon energy, proportionality constant, bandgap, and wavelength of the applied power, respectively [1].

Table S2: The high-resolution O1s spectra of oxygen-related peaks

Samples	Lattice oxygen	Hydroxyl groups	Adsorbed oxygen species
$SrTiO_3$	530 eV	531.6 eV	533.1 eV
1Cu:STO	529.5 eV	531.55 eV	533.2 eV
2Cu:STO	529.46 eV	531.4 eV	533.01 eV
5Cu:STO	529.6 eV	531.61 eV	533.22 eV

3-STEM, TEM, and HRTEM

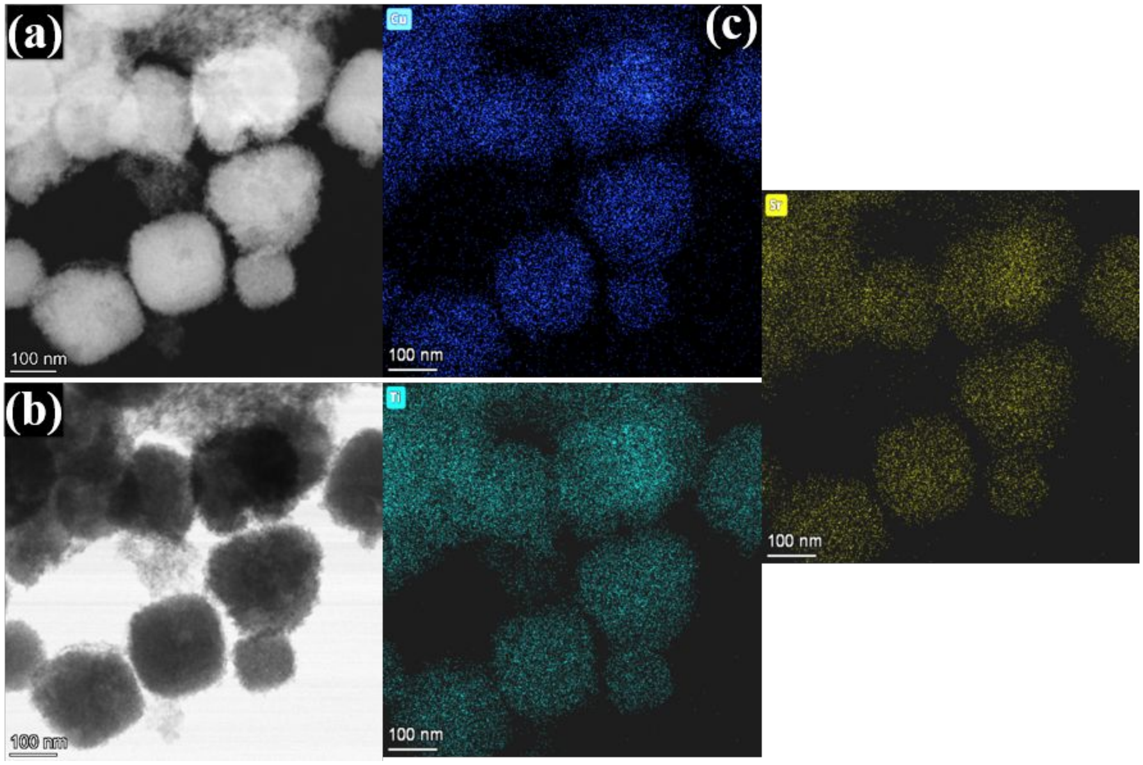

Figure S1 STEM images of the 5Cu:STO samples: (a) Dark-Field (DF); (b) Bright-Field (BF); (c) EDS elemental mapping (Ti in green, Sr in yellow, and Cu in blue).

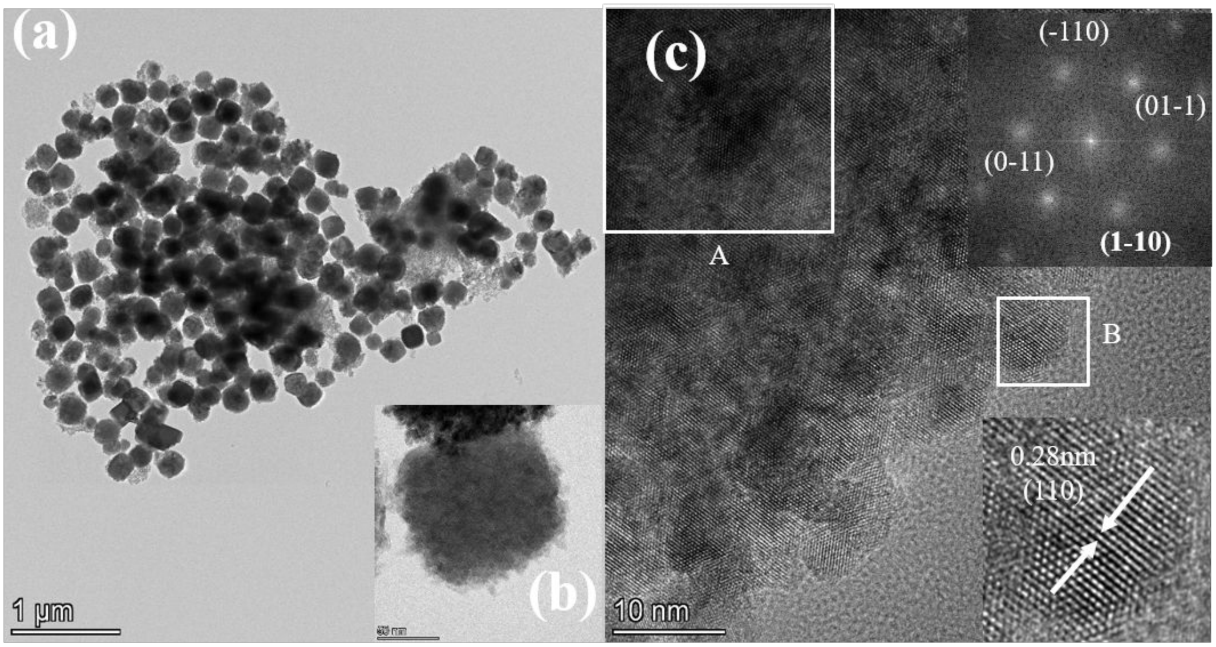

Figure S2 (a) TEM images of 5Cu:STO samples. (b) An expanded view illustrating an individual nanoparticle; (c) HRTEM image and the respective Fast Fourier transform (FFT) of region A (zone axis [111]) and an expanded view of region B.

Table S3: The Inductively Coupled Plasma Optical Emission Spectrometer (ICP OES).

Samples	The initial Molar of copper introduced (%)	Molar of Cu in the sample (%)	The Molar loss from the initial amount of copper (%)
(1Cu:STO)	0.395	0.093	76.65
(2Cu:STO)	0.69	0.123	82.17
(5Cu:STO)	1.73	0.442	74.45

Samples	Sr g/100g	Ti g/100g	Sr/Ti g/100g
(1Cu:STO)	33.8	29.3	1.15
(2Cu:STO)	30.3	28.1	1.07
(5Cu:STO)	31.8	28.3	1.12

4. Control experiments

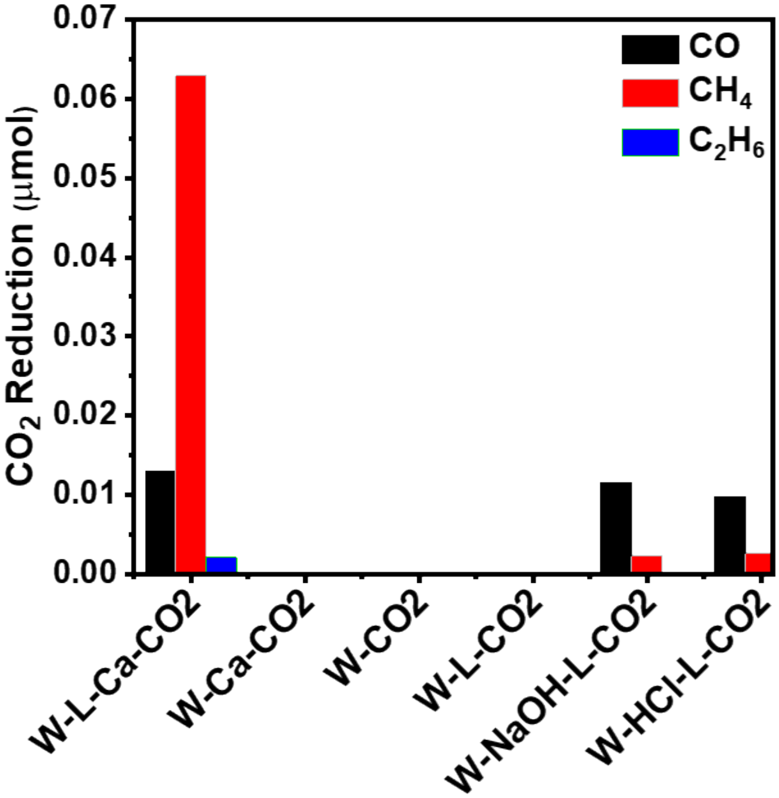

Figure S3 The control experiments over 2Cu:STO. The letters W, L, and Ca represent water, light, and catalyst, respectively.

Table S4: Comparison of CO_2 reduction production rate on $SrTiO_3$ with reported relevant catalysts.

Catalysts	Light source	Quantity/ Scavenger	Rate Production	Ref
$SrTiO_3$ doped Cu	Xe lamp (300-W) calibrated to 500 mW cm^{-2}	10 mg in 30ml of water	1.5 µmol g^{-1} $h^{-1}$of CH_4	This work
Fe_2TiO_5/ CuO	Xe lamp (300-W) calibrated to 800 mW cm^{-2}	25 mg in 50 mL of water with 0.1 mol/ L of $NaHCO_3$	0.5 µmol g^{-1} h^{-1}	[2]
$CuSrTiO_3$	Xe lamp (300-W)	20 mg of photocatalyst and 20 mL	6.96 µmol g^{-1} h^{-1} (methanol), CH_4 lower than 1µmol g^{-1} h^{-1}	[3]
Ti-H on hydrogenated $SrTiO_3$	ultraviolet lamps	10 mg/5ml water	6.4 µmol g^{-1} h^{-1}(C_2H_2) 1.95 µmol g^{-1} h^{-1}(CH_4)	[4]
$SrTiO_3$-Pt	Xe lamp (300 W)	50mg photocatalyst, aqueous triethanolamine (100 mL, 10 vol%)	4.39 µmol g^{-1} $h^{-1}$of CH_4	[5]

ZnO <26 nm_NaOH	Mercury lamp TQ 150 Z3 (150 W)	200mg/500ml water	4.04 μmol/g/dm^3 of CH_4	6

5. Photocatalytic CO_2 Reduction and Stability

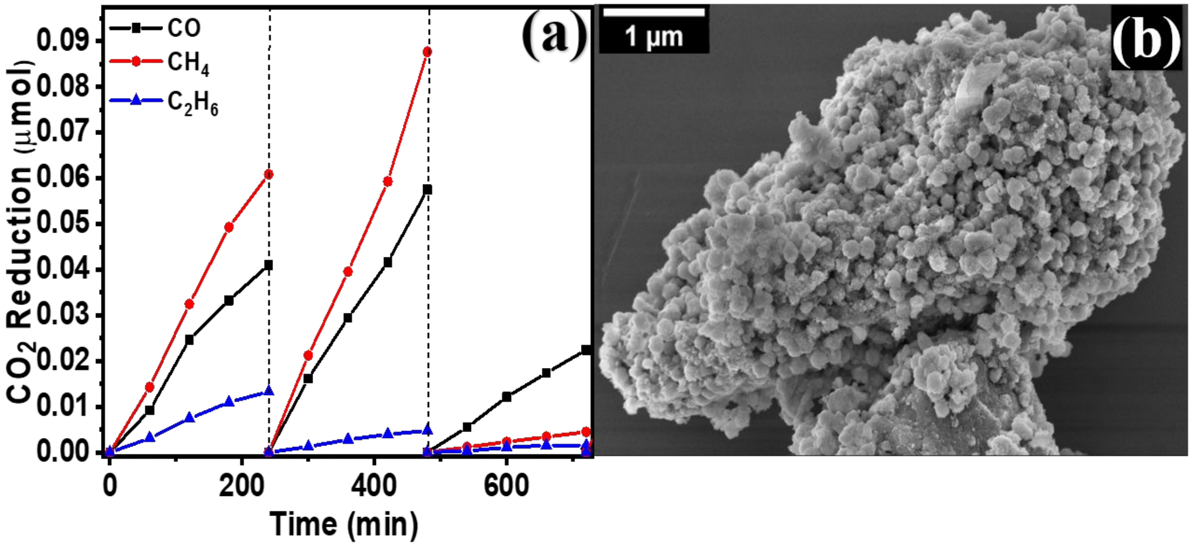

Figure S4 (a) Photocatalytic CO_2 reduction recyclability of 2Cu:STO with pH 8.3 controlled by NaOH, and (b) the FESEM after the recyclability of pH 8.3 controlled.

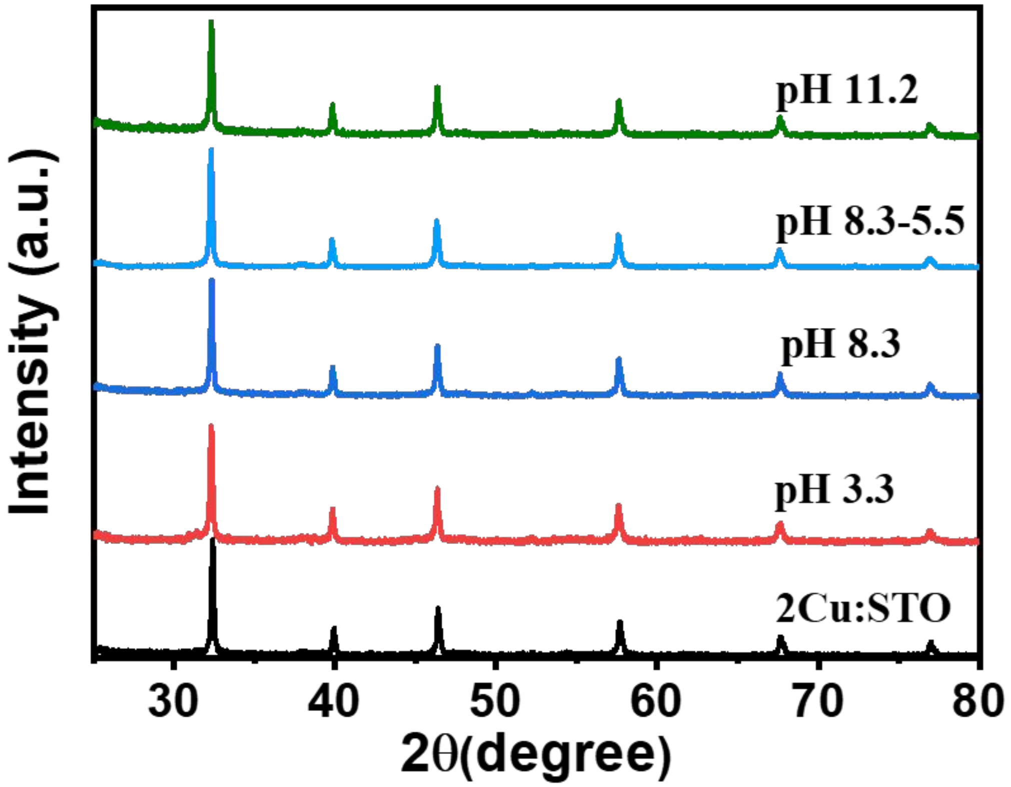

Figure S5 XRD after the stability test of 2Cu:STO.

6. Electrochemical Impedance

Electrochemical impedance spectroscopy (EIS) was conducted under illumination with the use of a 1.5 AM filter to further study the impact of the Cu doping (0.01, 0.02, and 0.05M) on the charge transfer properties of $SrTiO_3$, as shown in Figure S6a. The EIS plot shows that pure $SrTiO_3$ corresponds to a large semicircle, which suggests a high charge transfer resistance (Figure S6b). Conversely, the 2Cu:STO sample corresponds to a small semicircle, suggesting a lower charge transfer resistance.

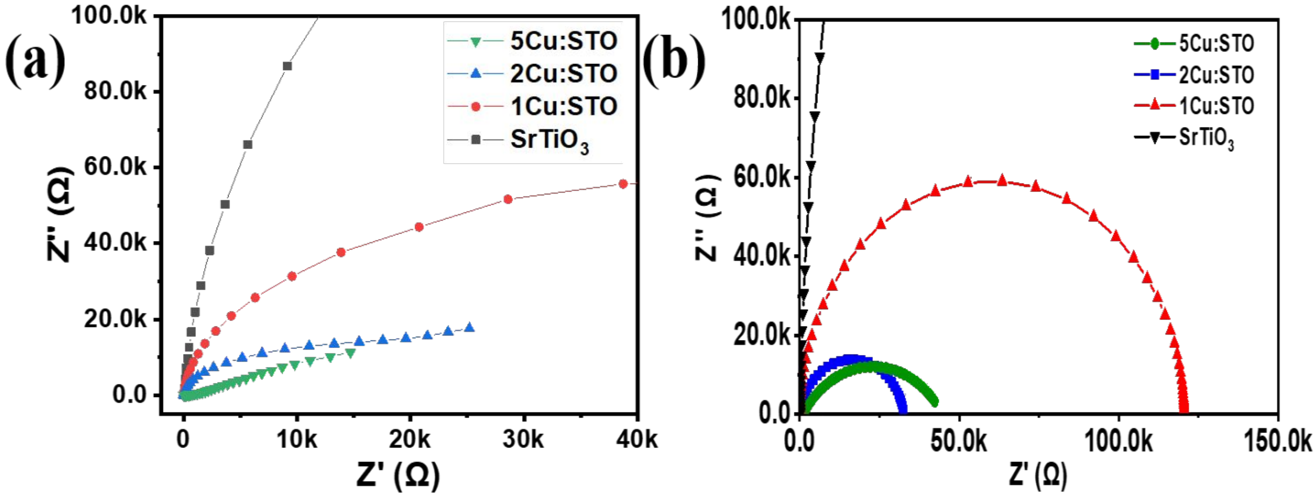

Figure S6 (a) Nyquist plots from EIS and (b) the fitting simulation of Nyquist plots of pure $SrTiO_3$ and Cu doped with Cu:STO.

References:

(1) Bajiri, M. A.; Hezam, A.; Namratha, K.; Al-Maswari, B. M.; BhojyaNaik, H. S.; Byrappa, K.; Al-Zaqri, N.; Alsalme, A.; Alasmari, R. Non-Noble Metallic Cu with Three Different Roles in a Cu Doped ZnO/Cu/GC 3 N 4 Heterostructure for Enhanced Z-Scheme Photocatalytic Activity. *New J. Chem.* **2021**, *45* (30), 13499–13511.

(2) dos Santos, G. H. C.; Gonçalves, R. V; Rossi, L. M. Photocatalytic CO2 Reduction to CH4 in Continuous Flow Reactor Using Fe2TiO5 Enhanced by Magnetron Sputtering-Deposited

CuO Nanoparticles Cocatalyst. *Mater. Lett.* **2024**, 136891.

(3) Ding, J.; Qiu, C.; Zhang, Z.; Qu, Q.; Fang, C.; Zhang, J.; Guan, G.; Wan, H. Enhanced Photocatalytic CO2 Reduction to Methanol by Modulating Valence States of Copper in CuSrTiO3. *Sep. Purif. Technol.* **2024**, *349*, 127876.

(4) Xu, H.; Wang, Z.; Liao, H.; Li, D.; Shen, J.; Long, J.; Dai, W.; Wang, X.; Zhang, Z. Proximity of Defects and Ti-H on Hydrogenated SrTiO3 Mediated Photocatalytic Reduction of CO2 to C2H2. *Appl. Catal. B Environ.* **2023**, *336*, 122935.

(5) Wei, H.; Cai, J.; Zhang, Y.; Zhang, X.; Baranova, E. A.; Cui, J.; Wang, Y.; Shu, X.; Qin, Y.; Liu, J. Synthesis of SrTiO 3 Submicron Cubes with Simultaneous and Competitive Photocatalytic Activity for H 2 O Splitting and CO 2 Reduction. *RSC Adv.* **2020**, *10* (70), 42619–42627.

(6) Kusiak-Nejman, E.; Ćmielewska, K.; Ekiert, E.; Wanag, A.; Kapica-Kozar, J.; Pełech, I.; Narkiewicz, U.; Morawski, A. W. The Comparison of Photocatalytic Reduction of CO2 on ZnO Photocatalysts at Slightly Acidic and Alkaline PH towards the Formation of Valuable Products. *Catal. Today* **2024**, *432*, 114599.
